# Remotely Sensed Rice Yield Prediction Using Multi-Temporal NDVI Data Derived from NOAA's-AVHRR

**DOI:** 10.1371/journal.pone.0070816

**Published:** 2013-08-13

**Authors:** Jingfeng Huang, Xiuzhen Wang, Xinxing Li, Hanqin Tian, Zhuokun Pan

**Affiliations:** 1 Institute of Agricultural Remote Sensing & Information Application, Zijingang Campus, Zhejiang University, Hangzhou, China; 2 China Ministry of Education Key Laboratory of Environmental Remediation and Ecological Health, Zhejiang University, Hangzhou, China; 3 Key Laboratory of Agricultural Remote Sensing and Information System, Zhejiang Province, China; 4 Institute of Remote Sensing and Earth Sciences, Hangzhou Normal University, Hangzhou, China; 5 International Center for Climate and Global Change Research, Auburn University, Auburn, Alabama, United States of America; 6 Ecosystem Dynamics and Global Ecology (EDGE) Laboratory, School of Forestry and Wildlife Sciences, Auburn University, Auburn, Alabama, United States of America; National Rice Research Center, United States of America

## Abstract

Grain-yield prediction using remotely sensed data have been intensively studied in wheat and maize, but such information is limited in rice, barley, oats and soybeans. The present study proposes a new framework for rice-yield prediction, which eliminates the influence of the technology development, fertilizer application, and management improvement and can be used for the development and implementation of provincial rice-yield predictions. The technique requires the collection of remotely sensed data over an adequate time frame and a corresponding record of the region's crop yields. Longer normalized-difference-vegetation-index (NDVI) time series are preferable to shorter ones for the purposes of rice-yield prediction because the well-contrasted seasons in a longer time series provide the opportunity to build regression models with a wide application range. A regression analysis of the yield versus the year indicated an annual gain in the rice yield of 50 to 128 kg ha^−1^. Stepwise regression models for the remotely sensed rice-yield predictions have been developed for five typical rice-growing provinces in China. The prediction models for the remotely sensed rice yield indicated that the influences of the NDVIs on the rice yield were always positive. The association between the predicted and observed rice yields was highly significant without obvious outliers from 1982 to 2004. Independent validation found that the overall relative error is approximately 5.82%, and a majority of the relative errors were less than 5% in 2005 and 2006, depending on the study area. The proposed models can be used in an operational context to predict rice yields at the provincial level in China. The methodologies described in the present paper can be applied to any crop for which a sufficient time series of NDVI data and the corresponding historical yield information are available, as long as the historical yield increases significantly.

## Introduction

Paddy rice is one of the most important and widely grown crops in China. The total paddy-rice production in 2009 reached 195.1 million tons, and it accounted for 40.5% of the total grain production in China (481.563 million tons) [1]. Timely, objective and quantitative information regarding to paddy-rice yield can provide important information for government agencies and producers that can be used for planning harvest, storage and marketing activities. Therefore, paddy-rice-yield prediction is important for the food security of China and is considered to be one of the most challenging tasks in agricultural research [2]. The traditional approach of crop-yield forecasting, the use of ground-based data collection is expensive, time-consuming, labor-intensive, and often difficult [3]. Crop-yield prediction using remotely sensed data has already represented a very active field of research and application [3]–[5]. Notable advances in remote-sensing technology over the last several decades are now providing scientists with valuable information for yield and production forecast. Time series of normalized-difference-vegetation-index (NDVI), derived from the satellite data, have been used for crop-yield predictions since the 1980's. Most of the studies that related NDVI measurements to crop yield have been concentrated on staple crops such as wheat [4], [6]–[38] and maize [3], [13], [18], [20], [21], [24], [29], [39]–[49] and rice [2], [15], [37], [44], [50]–[52]. Many researchers have also found that NDVI variables are very good at grain yield predictors of millet [53]–[57], sorghum [24], [56], [58], [59], barley [19], [24], [29], [60], [61], soybean [3], [24], [62], [63], ground nut [54], [59], sugar beet [29], alfalfa [29], rye [29], pea [19], [29], and canola [19] (Literature review was summarized in Table 1). However, remotely sensed yield prediction appears limited in rice.

**Table 1:** Relevant literatures that linked with crop yield forecast using remotely sensed data literatures are sorted according to the crop types.

Crop	reference
**wheat**	MacDonald et al., 1980; Rudorff et al., 1991; , Bullock, 1992; Benedetti et al., 1993; Gupta et al., 1993; Benedetti et al., 1993; Cheng, 1994; Dubey et al., 1994; Sridhar et al., 1994; Doraiswamy et al., 1995, 2003; Smith et al., 1995; Hochheim et al., 1998; Huang et al., 1999; Maselli et al., 2001; Boken et al., 2002; Labus et al., 2002; Manjunath et al., 2002; Mika et al., 2002; Bastiaanssen, et al., 2003; Kalubarme et al., 2003; Ferencz et al., 2004; Zhang et al., 2004; Kastensa et al., 2005; Mo et al., 2005; Wang et al., 2005; Patel et al., 2006; Ren et al., 2006; Moriondo et al., 2007; Prasad et al., 2007; Balaghi et al., 2008; Ren et al., 2008; Wall et al., 2008; Schut et al., 2009; Becker-Reshef et al., 2010; Mkhabela et al., 2011
**maize**	Quarmby et al., 1993; Hayes et al., 1996; Unganai et al., 1998; Lewis et al., 1998; Lee et al., 1999; Reynolds et al., 2000; Seiler et al., 2000; Maselli et al.,2001; Mika et al., 2002; Wannebo et al., 2003; Ferencz et al., 2004; Kastensa et al., 2005; Mkhabela et al., 2005; Mo et al., 2005; Prasad et al., 2006; Rojas, 2007; Ren, et al., 2008; Funk et al., 2009
**millet**	Rasmussen, 1992, 1997, 1998; Groten, 1993; Maselli et al.,2000
**sorghum**	Potdar, 1993; Fuller, 1998; Maselli et al., 2000; Kastensa et al., 2005
**barley**	Wendroth et al., 2003; Ferencz et al., 2004; Kastensa et al., 2005; Weissteiner et al., 2005; Mkhabela et al., 2011
**soybean**	Liu et al., 2002; Kastensa et al., 2005; Prasad et al., 2006; Esquerdo et al., 2011
**ground nut**	Rasmussen, 1997; Fuller, 1998
**sugar beet**	*Ferencz et al., 2004*
**alfalfa**	*Ferencz et al., 2004*
**rye**	Ferencz et al., 2004
**pea**	Ferencz et al., 2004; Mkhabela et al., 2011
**canola**	Mkhabela et al., 2011
**rice**	Tennakoon et al., 1992; Quarmby et al., 1993; Huang et al., 2002; Wang et al., 2002; Bastiaanssen, et al., 2003; Prasad et al., 2007; Huang et al., 2010

Different methods have been developed to predict crop yields using remotely sensed data, and the most common approach is, by generating regression model, to develop direct empirical relationships between the NDVI measurements and the crop yield [15], [19], [45], [57]. These approaches assume that measures of the photosynthetic capacity from spectral-vegetation indices are directly related to crop yield. This assumption is used because many of the conditions that affect crop growth, development and ultimately yield could be captured through spectra measurements such as the NDVI [64]. By using long-term historical-yield data as a dependent variable and remotely sensed data as an independent variable, a statistical regression function was generated to perform crop-yield predictions, whereas the actual crop yields depend on many more factors than the presence of spectral-vegetation indices [37]. Tilman et al. [65] noted that increased yields in cereal are mainly the result of greater inputs of fertilizer, water and pesticides, new crop species, and the improvement of management over the last decades. For all developing countries, modern varieties accounted for 21% of the growth in crop yields during the early Green Revolution period [66]. In Asia, rice production has more than doubled as a result of the expansion of cultivated area, the adoption of modern cultivars, increased investments in irrigation, and an increased use of fertilizer over the past 4 decades [67]. Hafner [68] found that linear growth has been the most common trend in maize, rice, and wheat yields for 188 nations over the past 40 years. This scenario also occurs in China. Although the inter-annual variability of NDVI (probably due to unexpected weather conditions or disasters) can reveal crop yield fluctuations [19], [59]; however, remotely sensed-NDVI cannot detect those human-induced factors that resulted in increase of rice yield. Therefore, to monitor and predict crop-yield cannot use NDVI measurements solely.

For unit-yield estimation, using one simple regression function (usually known as: Y = a+b * NDVI) would be incompatible as the advance of years, because simple regression would be likely neglect those man-induced factors in yield increase. However, few studies have analyzed the time trends of crop yields, which reflect the influence of technology development, fertilizer application, and management improvement. Moreover, the regression model between statistical data and NDVI cannot be extendable [19], [45] because cropping system and rice yield level is natural condition-dependent in China.

In consideration of social factors and regional differences for remotely sensed crop yield estimation in China, the objective of the present paper was to develop a methodological framework that may be adopted for the regional-, national- and international-scale prediction of crop yields. This methodology was based on a time series analysis of historical-yield information. Paddy rice was chosen to test the proposed methodology. To accomplish this objective, we needed to: (1) geographically regionalize rice cultivation area for remotely sensed monitoring; (2) analyze the historical trends in the grain yield of rice; (3) decompose the remotely sensed yield of rice from the long-term historical data; (4) select the optimal predictors, based on a correlation analysis between the remotely sensed yield and the AVHRR-derived NDVIs; (5) construct prediction models for rice yield; and (6) evaluate the potential for rice-grain-yield prediction in China using AVHRR NDVI data as predictors.

## Materials and Methodology

### 2.1. The Remote-Sensing dataset

The research presented in this paper relies on a time series of AVHRR NDVI composite imagery from July 1981 to December 2006, derived from the National Oceanic and Atmospheric Administration's (NOAA) series of Advanced Very High Resolution Radiometer (AVHRR) instruments, with a spatial resolution of 8 km, by the NASA Global Inventory Monitoring and Modeling Systems (GIMMS) group at the Laboratory for Terrestrial Physics. There are two 15-day composites per month: the first (15a) is a maximum value composite from the first day to 15^th^of the month; and the 15b composite is from days 16 till the end of the month. All data are available from the University of Maryland Global Land Cover Facility (http://glcf.umiacs.umd.edu/data/gimms/).

Pinzon et al. [69] and Tucker et al. [70] described in detail how the GIMMS data set was developed. A number of improvements have been made on the GIMMS NDVI database, with respect to previous NDVI data sets, including corrections for: (1) sensor degradation; (2) inter-sensor differences; (3) solar-illumination angle and sensor-view angle effects due to satellite drift; (4) volcanic stratospheric aerosol corrections for 1982–1984 and 1991–1994; (5) missing data in the Northern Hemisphere during winter, using interpolation; and (6) short-term atmospheric aerosol effects, atmospheric water-vapor effects, and cloud-cover physics [69], [70]. This data set is considered to be the most accurate, long-term AVHRR data record [71]. By comparing these data to new, improved coarse-resolution remotely sensed data from SPOT Vegetation instrument and MODIS instruments, recent study confirmed its suitability for long-term vegetation studies [72].

### 2.2. NDVI Variables

A large number of studies found a close relationship between crop yields and NDVI variables. The theory is: the NDVI value presents the yield level corresponding to every single pixel. Therefore, a simple regression function can be explained the yield: yield  =  a*NDVI + b; then the total yield can be obtained by multiplying planting area. By literature review, previous studies suggest three types of NDVI variables: original NDVI [13], [23], [42], [63], cumulative NDVI [8], [23], [38], [42], [45], [63], [73], [74], and average NDVI [34], [45], [63]. The cumulative NDVI and the corresponding average NDVI for the same period were highly correlated because of the linear nature of the operations involved. Only the original NDVIs and the average NDVIs were selected as input data for the prediction models in the present paper.

NDVI variables around the time of the maximum are strongly correlated with final yields [31], [35], [75]. Specifically, the rice yield is most determined by crop conditions during the heading (i.e. peak phenological phase of growth); and yield-reflectance relationships are typically the strongest after mid-season. In contrast, NDVI value changes that occur outside of the rice-growing period maybe not positively related to yield [52]. These relationships within changes of NDVI value suggests that the NDVIs during the mid-to-late growing period should be a good indicator of rice yield; meanwhile this phenomenon provides an approach to discriminate rice planting area from remote sensing image. Therefore, the first step of this study was to extract the maximum NDVI during the rice-growth period (*NDVI_max_*) for each studied province from the remote sensing dataset from the year 1982 to 2006. The maximum NDVI is equal to the peak value of the seasonal NDVI profile. Then, six other original NDVIs were calculated: the first, second, third and fourth biweekly NDVIs prior to the NDVI_max_ (*NDVI_maxb4_*) and the first and second biweekly NDVIs after the NDVI_max_ (*NDVI_maxa2_*). These seven biweekly composites span 3 months of raw AVHRR imagery, corresponding to the rice-growth period. Focusing on the NDVI response during the rice-growth period helps to identify rice-specific vegetation changes.

Hochheim and Barber [27] also found that NDVI estimators with longer integration periods minimized variability in yield prediction. Therefore, based on the seven original NDVIs, twenty-one average NDVIs, clustered around the time of the peak NDVI, were calculated using a rigorous arithmetic mean framework (Table 2). In total, 28 NDVI variables were generated. They include all of the possible combinations of the original seven NDVIs.

**Table 2:** NVDI variables and their calculation formulas.

	NDVIs	Description of formulas
**1**	*NDVI_maxb1_*	the first biweekly NDVI before NDVI_max_
**2**	N*DVImaxb2*	the second biweekly NDVI before NDVI_max_
**3**	*NDVI_maxb3_*	the third biweekly NDVI before NDVI_max_
**4**	*NDVI_maxb4_*	the fourth biweekly NDVI before NDVI_max_
**5**	*NDVI_max_*	the maximum NDVI during the growth period
**6**	*NDVI_maxa1_*	the first biweekly NDVI after NDVI_max_
**7**	*NDVI_maxa2_*	the second biweekly NDVI after NDVI_max_
**8**	*mNDVI_maxb4-b3_*	(NDVI_maxb4_+ NDVI_maxb3_)/2
**9**	*mNDVI_maxb4-b2_*	(NDVI_maxb4_+ NDVI_maxb3_+ NDVI_maxb2_)/3
**10**	*MNDVI_maxb4-b1_*	(NDVI_maxb4_+ NDVI_maxb3_+ NDVI_maxb2_+ NDVI_maxb1_)/4
**11**	*mNDVI_maxb4-max_*	(NDVI_maxb4_+ NDVI_maxb3_+ NDVI_maxb2_+ NDVI_maxb1_+ NDVI_max_)/5
**12**	*mNDVI_maxb4-a1_*	(NDVI_maxb4_+ NDVI_maxb3_+ NDVI_maxb2_+ NDVI_maxb1_+ NDVI_max_+ NDVI_maxa1_)/6
**13**	*mNDVI_maxb4-a2_*	(NDVI_maxb4_+ NDVI_maxb3_+ NDVI_maxb2_+ NDVI_maxb1_+ NDVI_max_+ NDVI_maxa1_+ NDVI_maxa2_)/7
**14**	*mNDVI_maxb3-b2_*	(NDVI_maxb3_+ NDVI_maxb2_)/2
**15**	*mNDVI_maxb3-b1_*	(NDVI_maxb3_+ NDVI_maxb2_+ NDVI_maxb1_)/3
**16**	*mNDVI_maxb3-max_*	(NDVI_maxb3_+ NDVI_maxb2_+ NDVI_maxb1_+ NDVI_max_)/4
**17**	*mNDVI_maxb3-a1_*	(NDVI_maxb3_+ NDVI_maxb2_+ NDVI_maxb1_+ NDVI_max_+ NDVI_maxa1_)/5
**18**	*mNDVI_maxb3-a2_*	(NDVI_maxb3_+ NDVI_maxb2_+ NDVI_maxb1_+ NDVI_max_+ NDVI_maxa1_+ NDVI_maxa2_)/6
**19**	*mNDVI_maxb2-b1_*	(NDVI_maxb2_+ NDVI_maxb1_)/2
**20**	*mNDVI_maxb2-max_*	(NDVI_maxb2_+ NDVI_maxb1_+ NDVI_max_)/3
**21**	*mNDVI_maxb2-a1_*	(NDVI_maxb2_+ NDVI_maxb1_+ NDVI_max_+ NDVI_maxa1_)/4
**22**	*mNDVI_maxb2-a2_*	(NDVI_maxb2_+ NDVI_maxb1_+ NDVI_max_+ NDVI_maxa1_+ NDVI_maxa2_)/5
**23**	*mNDVI_maxb1-max_*	(NDVI_maxb1_+ NDVI_max_)/2
**24**	*mNDVI_maxb1-a1_*	(NDVI_maxb1_+ NDVI_max_+ NDVI_maxa1_)/3
**25**	*mNDVI_maxb1-a2_*	(NDVI_maxb1_+ NDVI_max_+ NDVI_maxa1_+ NDVI_maxa2_)/4
**26**	*mNDVI_max-a1_*	(NDVI_max_+ NDVI_maxa1_)/2
**27**	*mNDVI_max-a2_*	(NDVI_max_+ NDVI_maxa1_+ NDVI_maxa2_)/3
**28**	*mNDVI_maxa1-a2_*	(NDVI_maxa1_+ NDVI_maxa2_)/2

### 2.3. Official Statistical Data of Rice Yield

**Figure 1:**
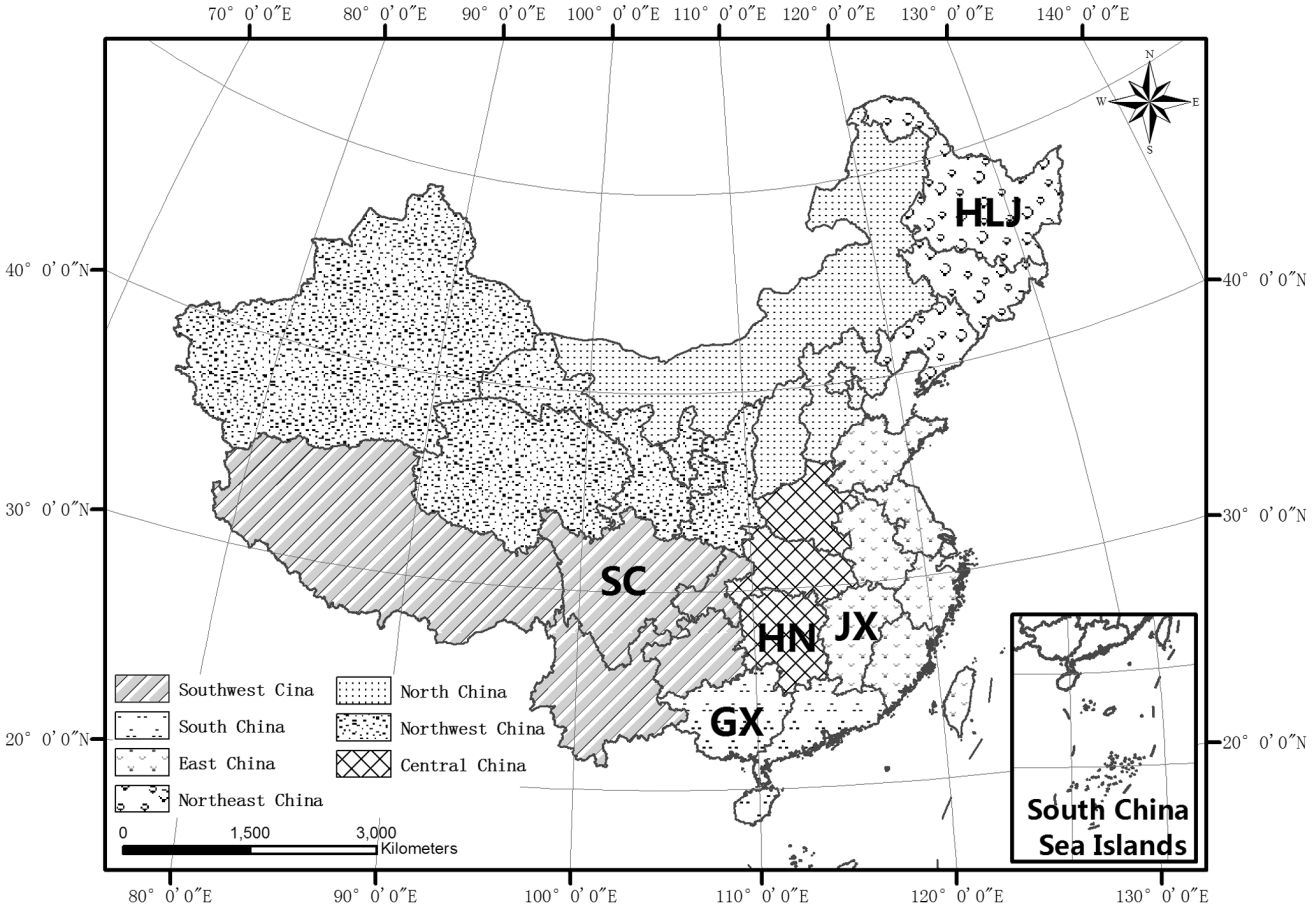
The locations of the study areas within Mainland China. Heilongjiang is designated by HLJ, Jiangxi by JX, Guangxi by GX, Sichuan by SC, and Hunan by HN.

Historical rice-yield data were acquired from the China Statistical Year Book by the National Bureau of Statistics of China (NBSC) from the years 1979 to 2009 [1]. The NBSC is the agency responsible for collecting and publishing agricultural statistics at the national and provincial levels. The NBSC crop statistics are based on data obtained from sub-province sample surveys and released in official documents. Customarily, Chinese provinces have been geographically grouped into 7 regions to present a spatial pattern for paddy rice planting area: Northeastern China (Heilongjiang, Jilin, and Liaoning), Northern China (Inner Mongolia, Hebei, Shanxi, Beijing, and Tianjin), Northwestern China (Ningxia, Shaanxi, Gansu, Qinghai, and Xinjiang), Central China (Henan, Hunan, and Hubei), Eastern China (Shandong, Jiangsu, Shanghai, Zhejiang, Anhui, and Jiangxi), Southwestern China (Chongqing, Sichuan, Guizhou, Yunnan, and Xizang), Southern China (Guangdong, Guangxi, Hainan) (see Figure 1). Unfortunately, rice planting area and yield information for Hong Kong- Macao-Taiwan areas was not available. According to NBSC crop statistical data (see Table 3), Eastern China was the region with the highest rice acreage and production levels (9808.60 kha and 64984.00 kt, respectively) in 2009. Central China ranked second in both rice acreage and production (6703.60 kha and 46215.00 kt, respectively). The third-largest rice cultivation and production area was Southwestern China (4448.10 kha and 31214.00 kt, respectively). Southern China and Northeastern China ranked fourth and fifth, respectively, in both rice acreage and production (4402.40 kha and 23499.00 kt; 3777.90 kha and 25855.00 kt, respectively). The total rice cultivation area in Eastern China, Central China, Southwestern China, Southern China, and Northeastern China is 29140.60 kha and accounts for 98.36% of the total rice cultivation area in the conterminous China. The total rice production in Eastern China, Central China, Southwestern China, Southern China, and Northeastern China was 191767.00 kt and accounted for 98.29% of the total rice production in the conterminous China in 2009. Northern China and Northwestern China constitute less than 2% of the national rice harvested area and production and were less important on a national scale in 2009.

**Table 3:** Planted area and production changes for rice between 1979 and 2009 for different regions in the conterminous China.

Regions	Area (Kha)				Production (Kt)			
	1979	% of China	2009	% of China	1979	% of China	2009	% of China
**Northeastern China**	841.73	2.49	3777.90	12.75	3860.00	2.69	25855.00	13.25
**Northern China**	264.07	0.78	204.40	0.69	1165.00	0.81	1343.00	0.69
**Northwestern China**	315.27	0.93	281.70	0.95	1305.00	0.91	1993.00	1.02
**Central China**	7639.13	22.55	6703.60	22.63	34260.00	23.83	46215.00	23.69
**Eastern China**	12926.33	38.16	9808.60	33.11	56230.00	39.12	64984.00	33.31
**Southwestern China**	4803.73	14.18	4448.10	15.01	21440.00	14.91	31214.00	16.00
**Southern China**	7082.40	20.91	4402.40	14.86	25490.00	17.73	23499.00	12.04
**Total**	33872.67	100.00	29626.70	100.00	143750.00	100.00	195103.00	100.00

### 2.4. Description of Study Area

We divided China into 7 regions together with 5 representative provinces selected to convey the information of paddy rice planting area: Heilongjiang (HLJ) in Northeastern China, Hunan (HN) in Central China, Jiangxi (JX) in Eastern China, Sichuan (SC) in Southwestern China, and Guangxi (GX) in Southern China. These provinces were selected as the study areas for the present research because these locations: (1) represented the typical cropping system in China, (2) are located in primary rice-production regions, and (3) are geographically and climatologically different (see Figure 1 and Table 4). The life span, cropping system, and planting schedule are all depend on regional hydro-thermal condition. The general information on life span, cropping system, total annual rainfall (mm), annual accumulated temperature (°C), area (kha), and production levels (kt) for the selected provinces is shown in Table 4. The total combined rice-cultivation area in Heilongjiang (HLJ), Hunan (HN), Jiangxi (JX), Sichuan (SC), and Guangxi (GX) is 13942.2 kha, and these regions accounted for 47.06% of the total rice-cultivation area in China in 2009. The total combined rice production in Heilongjiang (HLJ), Hunan (HN), Jiangxi (JX), Sichuan (SC), and Guangxi (GX) was 87251 kt and accounted for 44.72% of the total rice production in China in 2009. The time series of the NBSC province-level rice yields were used to train and develop the prediction models for these five provinces.

**Table 4:** General information on Rice cropping system, Life span, Total annual rainfall (mm), Annual accumulated temperature (≥10°C), Area (kha) and Production (kt) for the study areas.

Provinces	Climate region	Rice cropping system	Life span	Total annual rainfall (mm)	Annual accumulated temperature (≥10 °C)	Planting Area in 2009(kha)	Percent age of China (%)	Production in 2009 (kt)	Percenta ge of China (%)
**Heilongjiang** **(HLJ)**	Temperate continental monsoon climate	Single cropping	May – Oct	450–650	2000–3700	2460.80	8.31	15745.00	8.07
**Hunan** **(HN)**	Subtropical monsoon climate	Double cropping	Mar – Aug, Jun – Nov	1200–1700	4500–6500	4047.20	13.66	25786.00	13.22
**Jiangxi** **(JX)**	Subtropical monsoon climate	Double cropping	Mar – Aug, Jun - Nov	1300–2000	4500–6500	3282.10	11.08	19059.00	9.77
**Sichuan** **(SC)**	Subtropical humid climate	Single cropping	Mar - Aug	950–1200 (Sichuan Basin)	4000–6000 (Sichuan Basin)	2027.10	6.84	15202.00	7.79
**Guangxi** **(GX)**	Subtropical monsoon climate	Double cropping	Mar – Aug, Jun - Nov	1300–2000	5800–9300	2125.00	7.17	11459.00	5.87

### 2.5. Calibration of Rice-Yield Prediction Models

The gradual trend in yields is due to the influence of technological development, fertilizer application, and improved management on the rice cultivation. The results of this analysis suggest that the most common trend of rice yield is a linear growth. The province-specific intercepts account for spatial variations in rice management and soil quality; province-specific time trends account for yield growth due to technology gains. This indicates us the yield is composed from the intrinsic and extrinsic factors. Therefore, we decomposed the historical rice yield *Y* into the trend yield *Y_t_* and the remotely sensed yield *Y_RS_*, using the following equation:
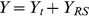
(1)
*Y_t_*, represents the component that is regulated by agricultural technology, including (1) the usual biological-chemical technologies (new varieties, fertilizers, herbicides, insecticides, etc.) and the mechanical technologies (machinery, equipment, etc.); (2) the management practices, which involve changes such as the timing of field operations and other practices which may or may not be involved in the purchase of new inputs. *Y_RS_* is defined as the component regulated by natural environmental conditions, such as temperature, precipitation, pests and disease; these environment factors can be detected by a remote sensor.

**Figure 2:**
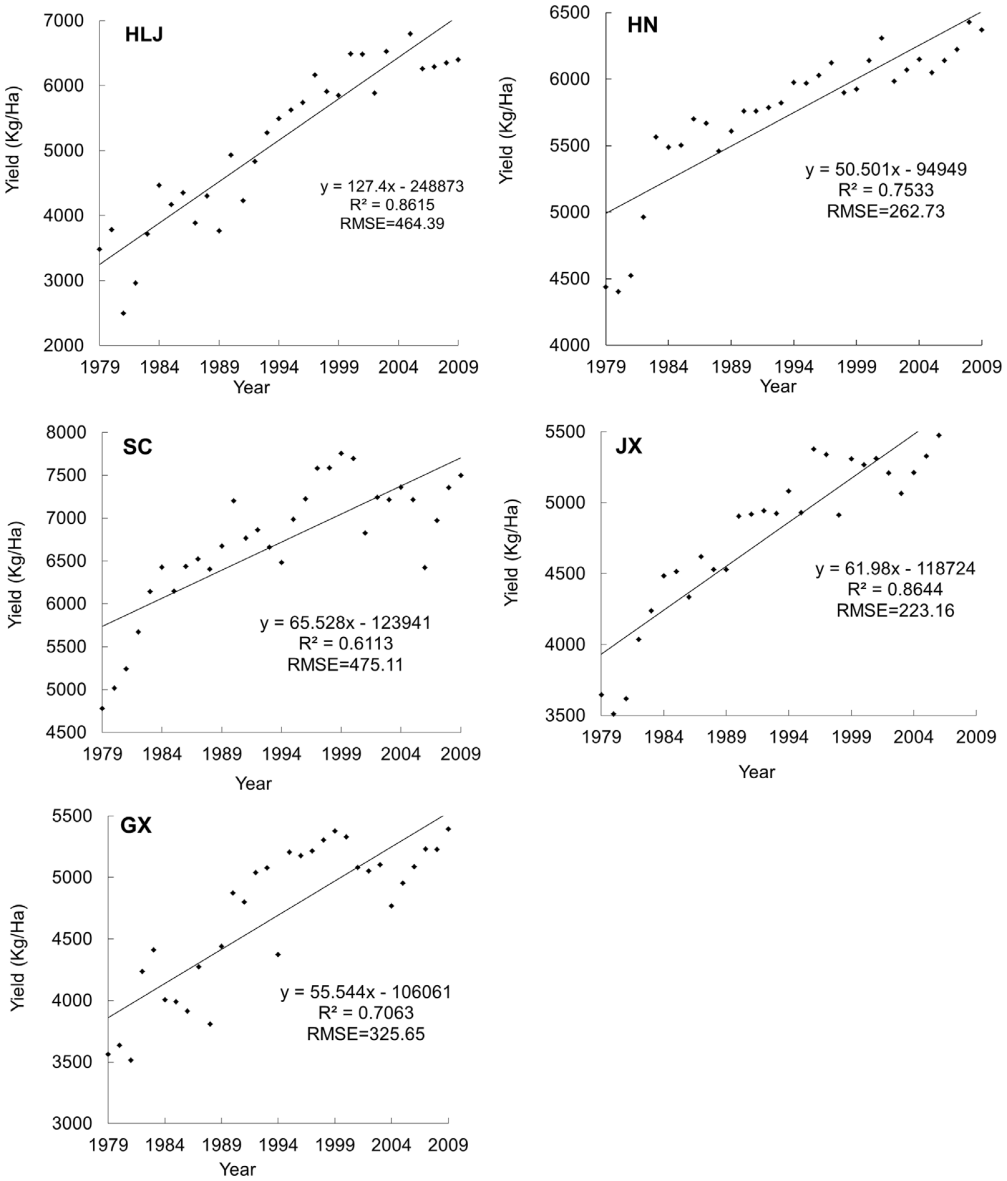
Rice yield trends for the provinces' of Heilongjiang (HLJ), Hunan (HN), Jiangxi (JX), Sichuan (SC) and Guangxi (GX) from 1979 to 2006.



To quantify past trends in yields, many different yield de-trend methods have been reported, including: least-squares regressions [76], [77], moving averages [78], [79], exponential algorithms [80], and polynomial regressions [81]. For rice-yield predictions in the present investigation, a linear regression model and a moving average are both generated to fit each separated provincial rice dataset (also see in Figure 2):

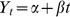
(2)where *Y_t_* is the trend yield in a given province during a given year (kg ha^−1^), *t* represents the year of harvest (the year 1979 was numeral 1979, 1980 was numeral 1980, etc., until 2009 was numeral 2009), α and β are the province-specific linear regression coefficients.

In our study, a moving average is used with historical crop-yield data to smooth out short-term fluctuations and highlight longer-term trends. Rice yields were de-trended using their deviations from the 5-year moving average. The mean changes in provincial historical rice yield (Y), the trend yield (Y_t_) and the remotely sensed yield (Y_RS_) were calculated for each period as an average of the changes from each single preceding year to the next by using a moving average method. Generally, the moving average method is used to calculate arithmetic mean of each five of the entire dataset: *y_i-n_, y_i-n+1_, …, y_i_, …., y_i+n_*. Such method has been usually employed in meteorological data analysis to remove the stochastic errors from long-time series of data. Hence, an algorithm for a 5-year moving average is as follows:
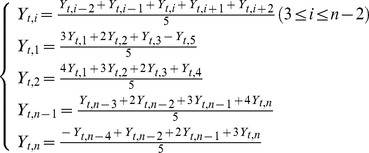
(3)Where *Y_t, i_* is the trend yield in a given province during a given year (kg ha^−1^); n represents the number of data points; *i* represents the year of the harvest (e.g. the year 1979 was numeral 1, 1980 was numeral 2, etc., until 2006 it should be numeral 31); *Y_t,1_* and *Y_t,2_* are the trend yields for the first two harvested years; then *Y_t, n-1_* and *Y_t, n_* are the trend yields for the last two harvested years within the 5 years.

Consequently, the trend yield *Y_t_* was obtained. To remove the technological influences, it is necessary to remove the yield trend to produce a new time series that is directly related to the NDVIs. We defined this new time series as the remotely sensed yield. According to Eq. (1), the remotely sensed yield can be calculated by the following equation:
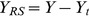
(4)


Next, correlation analysis was performed between the remotely sensed yield and the NDVI variables. The correlation coefficient is a measure of the strength and the direction of a linear relationship between two variables. The symbol *r* in Eq. (5) represents the samples' correlation coefficient; *x* and *y* represent the remotely sensed yield and the NDVI variables respectively; *n* is the number of data pairs.
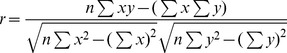
(5)


Statistical regression models are the most commonly used method for crop-yield prediction based on remotely sensed data [8], [36]. They do not require numerous inputs and can be performed directly; also because it requires little computing power and the selected variables are distinctive and non-overlapping. Therefore, each of the provincial *Y_RS_* and NDVI dataset was analyzed separately by means of stepwise regression techniques. These models were constructed via the ‘STEPWISE’ regression process which was available in software Statistical Product and Service Solutions (SPSS) 17.0 [82]. The probability significance thresholds for the entry and retention of candidate independent variables in the model were both set to α = 5%.

### 2.6. Evaluation of Rice-Yield Prediction Models

The rice-yield prediction models were evaluated using the following indicators:

Root mean square error (RMSE):
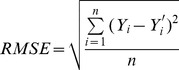
(6)


Coefficient of determination (R^2^):
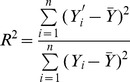
(7)


F-value (F):
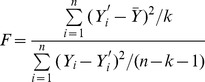
(8)


and relative error (RE):
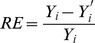
(9)


Together with the above, where *n* is the number of comparisons; *k* is the number of predictors; *Y_i_* is the statistical rice yield; 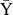 is the average rice yield, and 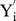 is the predicted yield.

## Results and Discussion

### 3.1. Rice Yield Trend Analysis

Figure 2 presents the evolution of the average rice-grain yield in Heilongjiang (HLJ), Jiangxi (JX), Guangxi (GX), Sichuan (SC), and Hunan (HN) from 1979 to 2009; according to their R-square and RMSE, all rice yields showed a visible and significant growth trend over time. Understanding the past rice-yield trends can help us to gauge the importance of the preprocessing procedure for rice-yield prediction using remotely sensed data. The statistical data of rice yield together with average yield growth trend from 1979 to 2009 in five provinces of China is summarized in Table 5.

**Table 5:** Trends in rice yield for five selected-provinces in China from 1979 to 2009.

Province	Yield in 1979 (kgha^−1^)	Yield in 2009(kgha^−1^)	Annual increase, 1979–2009 (kgha^−1^yr^−1^)
**Heilongjiang (HLJ)**	3480	6398.3	94.14
**Hunan (HN)**	4440	6371.3	62.30
**Jiangxi (JX)**	3645	5807	69.74
**Sichuan (SC)**	4777.5	7499.4	87.80
**Guangxi (GX)**	3562.5	5392.5	59.03

As analysis above (see Figure 2), the social input and advance of technology account for the linear trend of the rice-yield growth, whereas such human-induced factors could not be detected using remotely sensed data. To overcome this problem and make rice-yield prediction methods more robust and easily exportable, one possible strategy is to integrate remote-sensing data with the rice yield time series analysis. De-trending is necessary to properly identify the remote-sensible effects in these panel datasets. Therefore, before the rice-yield predicting models are established using remotely sensed variables as predictors, we suggest that the statistical yield should be decomposed into the trend yield and the remotely sensed yield, methodology was described in Section 2.5.

### 3.2. Correlation Coefficients between the Remotely Sensed Yield and NDVI Variables

The correlation coefficients between Y_RS_ and the NDVI variables for the rice-growth period from the fourth 15-day period before *NDVI_max_* (*NDVI_maxb4_*) to the second 15-day period after *NDVI_max_* (*NDVI_maxa2_*) for each of the studied provinces are summarized in Table 6. By comparing the correlation coefficients (Column 2 and 3 in Table 6), the *Y_RS_* that was de-trended by linear regression performed better than the Y_RS_ that was de-trended by a 5-year moving average against the NDVI variables.

**Table 6:** Correlation coefficient (R) between the remotely sensed yields and NDVI variables during the rice growth period.

Variables	the remotely sensed yields de-trended by linear models					the remotely sensed yields de-trended by 5-year moving average				
	HLJ	HN	JX	SC	GX	HLJ	HN	JX	SC	GX
**NDVI_maxb4_**	−0.02	−0.08	0.05	0.68**	0.24	−0.12	0.14	0.04	0.51**	0.54**
**NDVI_maxb3_**	−0.16	−0.02	0.14	0.73**	0.36	−0.21	0.13	0.10	**0.46** *	0.52**
**NDVI_maxb2_**	−0.08	0.38	0.21	0.57**	−0.14	−0.06	0.34	0.14	0.39	−0.30
**NDVI_maxb1_**	−0.06	0.56**	**0.42** *	0.32	0.19	−0.03	0.22	−0.04	0.16	0.09
**NDVI_max_**	0.13	0.60**	0.39	−0.06	−0.04	0.20	0.20	0.10	0.10	−0.26
**NDVI_maxa1_**	**0.42** *	0.62**	0.28	0.29	−0.01	0.35	0.27	−0.05	0.08	−0.28
**NDVI_maxa2_**	0.20	**0.49** *	0.32	−0.11	0.38	0.28	0.18	0.01	−0.22	0.39
**mNDVI_maxb4-b3_**	−0.08	−0.05	0.10	0.73**	0.31	−0.16	0.14	0.08	**0.50** *	0.57**
**mNDVI_maxb4-b2_**	−0.09	0.12	0.16	0.73**	0.19	−0.14	0.25	0.11	**0.50** *	0.32
**mNDVI_maxb4-b1_**	−0.08	0.25	0.26	0.66**	0.22	−0.13	0.28	0.08	**0.43** *	0.30
**mNDVI_maxb4-max_**	−0.07	0.33	0.29	0.64**	0.22	−0.11	0.30	0.09	**0.44** *	0.26
**mNDVI_maxb4-a1_**	0.09	**0.47** *	0.31	0.61**	0.18	0.03	0.33	0.07	0.39	0.12
**mNDVI_maxb4-a2_**	0.15	0.56**	0.33	0.54**	0.25	0.12	0.35	0.06	0.31	0.20
**mNDVI_maxb3-b2_**	−0.14	0.23	0.20	0.70**	0.10	−0.15	0.28	0.14	**0.46** *	0.06
**mNDVI_maxb3-b1_**	−0.12	0.37	0.32	0.60**	0.15	−0.13	0.30	0.09	0.38	0.08
**mNDVI_maxb3-max_**	−0.10	**0.45** *	0.35	0.58**	0.14	−0.09	0.31	0.10	0.38	0.02
**mNDVI_maxb3-a1_**	0.13	0.57**	0.35	0.55**	0.09	0.10	0.34	0.07	0.33	−0.10
**mNDVI_maxb3-a2_**	0.19	0.64**	0.37	**0.46** *	0.18	0.19	0.35	0.06	0.24	0.03
**mNDVI_maxb2-b1_**	−0.08	0.51**	0.35	**0.47** *	0.00	−0.05	0.33	0.07	0.29	−0.15
**mNDVI_maxb2-max_**	−0.03	0.59**	0.38	**0.44** *	−0.01	0.01	0.34	0.08	0.29	−0.21
**mNDVI_maxb2-a1_**	0.25	0.66**	0.37	**0.43** *	−0.01	0.23	0.34	0.04	0.25	−0.25
**mNDVI_maxb2-a2_**	0.27	0.69**	0.38	0.33	0.10	0.29	0.33	0.04	0.15	−0.09
**mNDVI_maxb1-max_**	0.02	0.64**	**0.46** *	0.25	0.13	0.07	0.24	0.01	0.17	−0.05
**mNDVI_maxb1-a1_**	0.34	0.69**	**0.42** *	0.30	0.06	0.31	0.28	−0.01	0.15	−0.19
**mNDVI_maxb1-a2_**	0.30	0.69**	**0.41** *	0.18	0.19	0.32	0.27	−0.01	0.02	0.01
**mNDVI_max-a1_**	**0.40** *	0.66**	0.35	0.23	−0.02	0.36	0.27	0.00	0.12	−0.31
**mNDVI_max-a2_**	0.33	0.66**	0.37	0.07	0.16	0.34	0.26	0.01	−0.06	−0.02
**mNDVI_maxa1-a2_**	0.32	0.62**	0.32	0.09	0.19	0.33	0.25	−0.02	−0.09	0.04

* significant at 0.05 level; ** significant at 0.01 level, n = 23.

The correlation coefficients between the Y_RS_ that were de-trended by linear regression and the NDVI variables were generally high in HN and SC. According to Table 6, for HN, the correlation coefficients were significant at the 0.01 level between the Y_RS_ that was de-trended by linear regression and *NDVI_maxb1_, NDVI_max_, NDVI_maxa1_, mNDVI_maxb4-a2_,mNDVI_maxb3-a1_, mNDVI_maxb3-a2_, mNDVI_maxb2-b1_, mNDVI_maxb2-max_, mNDVI_maxb2-a1_, mNDVI_maxb2-a2_, mNDVI_maxb1-max_, mNDVI_maxb1-a1_, mNDVI_maxb1-a2_, mNDVI_max-a1_, mNDVI_max-a2_,* and *mNDVI_maxa1-a2_*; the correlation coefficients were significant at the 0.05 level between the Y_RS_ that was de-trended by linear regression and *NDVI_maxa2_, mNDVI_maxb4-a1_,* and *mNDVI_maxb3-max_*. For SC, the correlation coefficients were significant at the 0.01 level between the Y_RS_ that was de-trended by linear regression and *NDVI_maxb4_, NDVI_maxb3_, NDVI_maxb2_,mNDVI_maxb4-b3_, mNDVI_maxb4-b2_, mNDVI_maxb4-b1_, mNDVI_maxb4-max_, mNDVI_maxb4-a1_, mNDVI_maxb4-a2_, mNDVI_maxb3-b2_, mNDVI_maxb3-b1_,* and *mNDVI_maxb3-max_, mNDVI_maxb3-a1_*; the correlation coefficients were significant at the 0.05 level between the Y_RS_ that was de-trended by linear regression and *mNDVI_maxb3-a2_, mNDVI_maxb2-b1_, mNDVI_maxb2-max_,* and *mNDVI_maxb2-a1_*. The highest correlation coefficient between the Y_RS_ that was de-trended by linear regression and the NDVI variables occurred in the second 15-day period after *NDVI_max_* (*NDVI_maxa2_*) and was significant at the 0.05 level for HLJ. The correlation coefficients between the Y_RS_ that was de-trended by linear regression and *NDVI_maxb1_, mNDVI_maxb1-max_, mNDVI_maxb1-a1_,* and *mNDVI_maxb1-a2_* were significant at the 0.05 level in JX. The correlation coefficients between the Y_RS_ that was de-trended by linear regression and the NDVI variables ranged from – 0.14 to 0.38 in GX.

The correlation coefficients between the *Y_RS_* that were de-trended by a 5-year moving average and the NDVI variables were generally low in HLJ, HN, and JX. For SC, the correlation coefficients were significant at the 0.01 level between the *Y_RS_* that was de-trended by a 5-year moving average and *NDVI_maxb4_*, and the correlation coefficients were significant at the 0.05 level between the *Y_RS_* that was de-trended by a 5-year moving average and *NDVI_maxb3_, mNDVI_maxb4-b3_, mNDVI_maxb4-b2_, mNDVI_maxb4-b1_, mNDVI_maxb4-max_,* and *mNDVI_maxb3-b2_*. The correlation coefficients were significant at the 0.01 level between the *Y_RS_* that was de-trended by a 5-year moving average and *NDVI_maxb4_*, *NDVI_maxb3_*, and *NDVI_maxb4-b3_*.

### 3.3. Remotely Sensed Yield-Prediction Models

Conclusions drawn in the yield-trend analysis and the correlation analysis between Y_RS_ and the NDVI variables encouraged us to attempt to build a simple remotely sensed yield-prediction model for rice based on the NDVI variables. According to the correlation coefficient result summarized in Table 6, the Y_RS_ values that were de-trended by linear regression were used as dependent variables in HLJ, HN, JX, and SC. The Y_RS_ values that were de-trended by a 5-year moving average were used as dependent variables in GX. The NDVIs were used as independent variables. These models were constructed through the ‘STEPWISE’ regression process in SPSS software. Each model contains variables using the data period from 1982 to 2004. The correlation coefficients of the selected models ranged from 0.42 to 0.92, and all models were significant at the 0.01 level, except for HLJ which is significant at the 0.05 level (see Table 7). This means that increases in NDVI during the rice-growth period are generally related to the final rice-grain yield. The influence of NDVI always had a positive impact on yield. These results are consistent with numerous previous studies [34], [36], [42], [75]. Data from 2005 to 2006 were used for model validation.

**Table 7:** Results of the stepwise regression models for remotely sensed rice yield using AVHRR-derived NDVI measures as independent variables.

Study areas	Model	R	F-test value	RMSE
**HLJ**	Y_RS_ = −849.158+0.137NDVI_maxa1_	0.42*	4.508	361.99
**HN**	Y_RS_ = −1240.690+0.229 mNDVI_maxb1-a2_	0.69**	19.342	114.57
**JX**	Y_RS_ = −1553.145+0.261 mNDVI_maxb1-max_	0.46**	5.689	166.38
**SC**	Y_RS_ = −1495.515+0.403 mNDVI_maxb4-b3_	0.73**	24.238	207.07
**GX**	Y_RS_ = −1832.285+1.138 mNDVI_maxb4-b3_ + 0.214NDVI_maxa2_ – 1.315 mNDVI_maxb4-b2_+0.307 mNDVI_maxb2-b1_	0.92**	25.103	87.70

R: multiple correlation coefficient.

* significant at 0.05 level; ** significant at 0.01 level.

### 3.4. Validation of Rice-Yield Prediction Models

**Figure 3:**
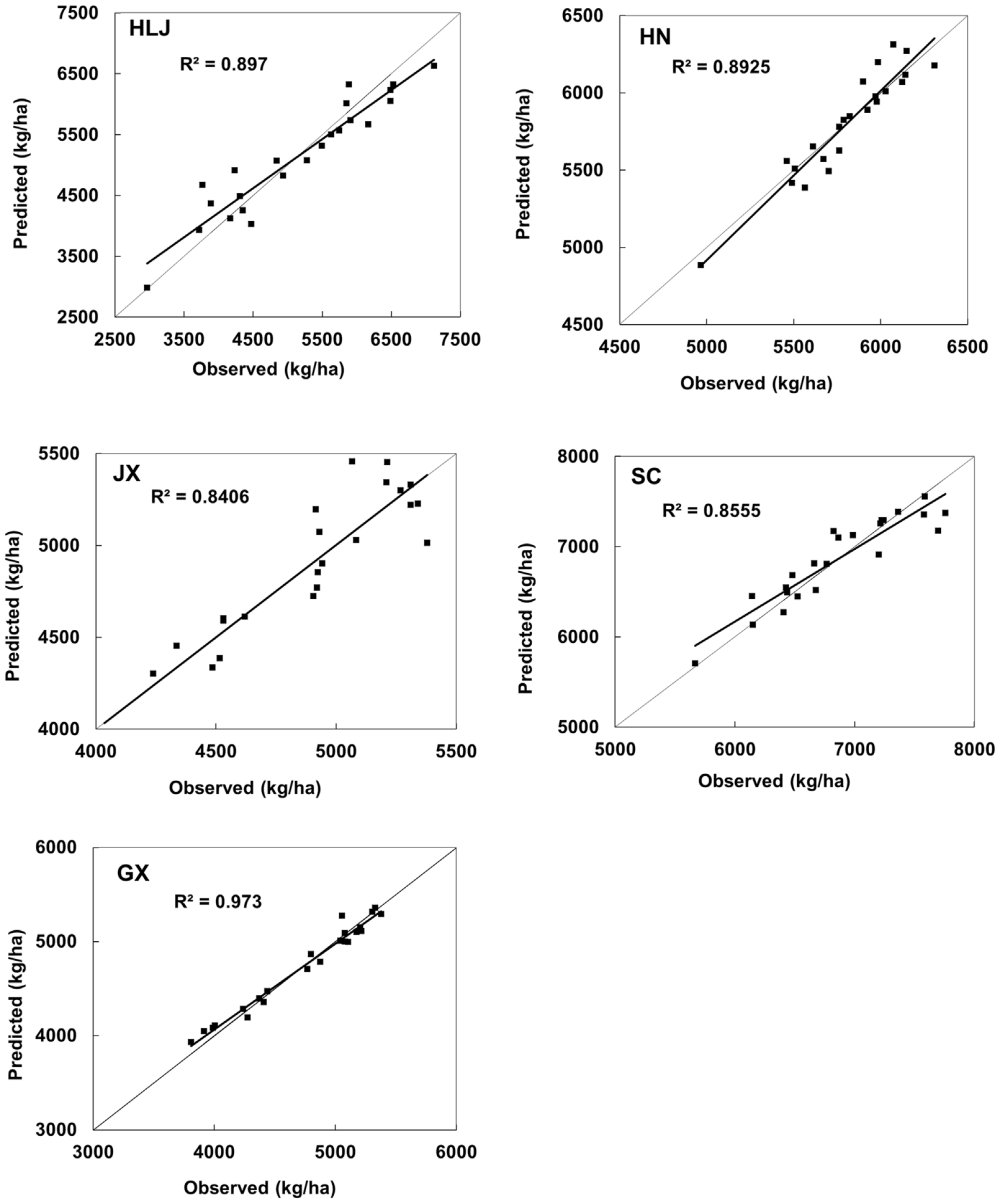
Observed versus predicted yields of rice (kg/ha) for the provinces of Heilongjiang (HLJ), Hunan (HN), Jiangxi (JX), Sichuan (SC) and Guangxi (GX) over the period 1982–2004.

The remotely sensed yield (*Y_RS_*) of rice was calculated using the NDVI variables required by each model described in Table 7. The final rice yield (*Y*) was the sum of the trend yield (*Y_t_*) and the remotely sensed yield (*Y_RS_*). Figure 3 shows a scatter plot of the predicted and observed final rice yields for HLJ, HN, JX, SC, and GX from 1982 to 2004, expressed in units of kilogram per hectare. The models performed well, showing a good similarity between the predicted values and the official statistical values in HLJ, HN, JX, SC, and GX from 1982 to 2004 and capturing the fluctuations of rice yields over time. The regression line between the predicted values and the observed values was close to the diagonal (intercept = 0, slope = 1), and the coefficients of determination for the five study areas ranged from 0.84 to 0.98, indicating that the reliability of the forecasts are very high.

The yield data for 2005 and 2006 were not included in the model construction and instead were used to evaluate the prediction models independently. These data provide independent estimates of the predictive power of the selected models (Table 8). The differences between the predicted values and the official statistical values were 5% or less in seven out of ten years. These results demonstrate the potential of a NDVI rice-yield estimate that is based on model calibration with historical data at the provincial level. However, it is noticeable that the predicted relative errors were greater than 10%, but less than 19% in both 2005 and 2006 for SC and in 2006 in HLJ when compared with the official statistical data. These error rates are likely due to a number of contamination sources that can confound the potential relationship between NDVIs and rice yield. For instance, cloud and atmospheric-moisture contamination can influence the NDVI signal. Vegetation signals from before or after the selected NDVIs can impact the final yield of rice.

**Table 8:** Observed and predicted rice yields (independent test).

Provinces	Year	Observed(kg/ha)	Predicted(kg/ha)	Relative Error (%)
**Heilongjiang (HLJ)**	2005	6795.7	6780.7	−0.22
	2006	6261.3	6897.8	10.17
**Hunan (HN)**	2005	6050.3	6337.5	4.75
	2006	6141.3	6441.2	4.88
**Jiangxi (JX)**	2005	5328.2	5545.9	4.09
	2006	5475.1	5634.9	2.92
**Sichuan (SC)**	2005	7213.0	8018.4	11.17
	2006	6420.7	7680.3	19.62
**Guangxi (GX)**	2005	4953.0	5028.98	1.53
	2006	5088.0	5053.44	−0.68

## Conclusion

This study focused on the obvious and important role that advance of technology plays in rice yields increase. The results of this analysis suggest that the most common trend of rice yields in China during the years 1979–2009 is a linear growth. In the light of rice-yield trend could not be detected directly by a satellite remote sensor therefore, yield de-trended analysis was necessary to properly identify the remote-sensible effects and obtain an accurate prediction for rice yield. Only with de-trending analysis could we interpret the NDVI's evolution as being mainly due to variations in the photosynthetic activity and growth conditions of rice and then predict the rice yield using NDVI variables.

The AVHRR-based indices explored in the present research were useful for the remotely sensed rice yield-prediction in major rice cultivation areas of China. This method allowed us to have a fine provincial estimate which satellite image could be difficult to obtain, or else a similar cost and a similar time frame data is easily available. However, it is cautious to restrict these analysis to those areas where the common trend of the crop yield is linear growth for the period considered.

The two steps for de-trending the statistical yield to obtain new time series, that are the trend yield (*Y_t_*) and the remotely sensed yield (*Y_RS_*); And by constructing the prediction models of *Y_RS_* using NDVI variables enabled the development of a robust, simple, remotely sensed data-based model that was applicable at the provincial level in China. We believe the approach introduced here has a wide applicability to other rice-producing countries as well as other crops, such as wheat and corn.

More empirical studies should be performed on the use of AVHRR-derived NDVI time series as predictors for crop yield to enhance the understanding its forecasting capacity and limitations, and to validate the methods of remotely sensed yield estimation further. A future study should also include the application of a longer AVHRR NDVI time series in combination with other data sets such as SPOT-VEG, MODIS and SeaWiFS, especially in the event of one of these dataset's unexpected absence.

## References

[1] National Bureau of Statistics of China (2010) China Statistical Year Book. Beijing: China Statistical Press.

[2] Wang RC, Huang JF (2002) Rice yield estimation using remote sensing data. Beijing: China Agriculture Press. 287 p. (in Chinese with English abstract).

[3] Prasad AK, Chai L, Singh RP, Kafatos M (2006) Crop yield estimation model for Iowa using remote sensing and surface parameters. International Journal of Applied Earth Observation and Geoinformation 8: 26–33.

[4] Manjunath KR, Potdar MB, Purohit NL (2002) Large area operational wheat yield model development and validation based on spectral and meteorological data. International Journal of Remote Sensing 23: 3023–3038.

[5] Salazar L, Kogan F, Roytman L (2007) Use of remote sensing data for estimation of winter wheat yield in the United States. International Journal of Remote Sensing 28: 3795–3811.

[6] Zhang F, Wu BF, Luo ZM (2004) Winter wheat yield predicting for America using remote sensing data. Journal of remote sensing 8: 611–617 (in Chinese with English abstract)..

[7] Wang CY, Lin WP (2005) Winter wheat yield estimation based on MODIS EVI. Transactions of the Chinese Society of Agricultural Engineering 21: 90–94 (in Chinese with English abstract)..

[8] Wall L, Larocque D, Leger PM (2008) The early explanatory power of NDVI in crop yield modelling. International Journal of Remote Sensing 29: 2211–2225.

[9] Sridhar VN, Dadhwal VK, Chaudhari KN, Sharma R, Bairagi GD, et al (1994) Wheat production forecasting for a predominantly unirrigated region in Madhya-Pradesh (India). International Journal of Remote Sensing 15: 1307–1316.

[10] Smith RCG, Adams J, Stephens DJ, Hick PT (1995) Forecasting wheat yield in a Mediterranean-type environment from the NOAA satellite. Australian Journal of Agricultural Research 46: 113–125.

[11] Schut AGT, Stephens DJ, Stovold RGH, Adams M, Craig RL (2009) Improved wheat yield and production forecasting with a moisture stress index, AVHRR and MODIS data. Crop & Pasture Science 60: 60–70.

[12] Rudorff BFT, Batista GT (1991) Wheat yield estimation at the farm level using TM-Landsat and agrometeorological data. International Journal of Remote Sensing 12: 2477–2484.

[13] Ren JQ, Chen ZX, Zhou QB, Tang HJ (2008) Regional yield estimation for winter wheat with MODIS-NDVI data in Shandong, China. International Journal of Applied Earth Observation and Geoinformation 10: 403–413.

[14] Ren JQ, Chen ZX, Tang HJ (2006) Regional scale remote sensing-based yield estimation of winter wheat by using MODIS-NDVI data: A case study of Jining City in Shandong Province. Chinese Journal of Applied Ecology 17: 2371–2375 (in Chinese with English abstract).. 17330482

[15] Prasad AK, Singh RP, Tare V, Kafatos M (2007) Use of vegetation index and meteorological parameters for the prediction of crop yield in India. International Journal of Remote Sensing 28: 5207–5235.

[16] Patel NR, Bhattacharjee B, Mohammed AJ, Tanupriya B, Saha SK (2006) Remote sensing of regional yield assessment of wheat in Haryana, India. International Journal of Remote Sensing 27: 4071–4090.

[17] Moriondo M, Maselli F, Bindi M (2007) A simple model of regional wheat yield based on NDVI data. European Journal of Agronomy 26: 266–274.

[18] Mo X, Liu S, Lin Z, Xu Y, Xiang Y, et al (2005) Prediction of crop yield, water consumption and water use efficiency with a SVAT-crop growth model using remotely sensed data on the North China Plain. Ecological Modelling 183: 301–322.

[19] Mkhabela MS, Bullock P, Raj S, Wang S, Yang Y (2011) Crop yield forecasting on the Canadian Prairies using MODIS NDVI data. Agricultural and Forest Meteorology 151: 385–393.

[20] Mika J, Kerenyi J, Rimoczi-Paal A, Merza A, Szinell C, et al. (2002) On correlation of maize and wheat yield with NDVI: Example of Hungary (1985–1998). In: Fellous JL, LeMarshall JF, Choudhury BJ, Menenti M, Paxton LJ et al.., editors. Earth's Atmosphere, Ocean and Surface Studies. 2399–2404.

[21] Maselli F, Rembold F (2001) Analysis of GAC NDVI data for cropland identification and yield forecasting in Mediterranean African countries. Photogrammetric Engineering and Remote Sensing 67: 593–602.

[22] MacDonald R, Hall F (1980) Global crop forecasting. Science 208: 670. 17771086 10.1126/science.208.4445.670

[23] Labus MP, Nielsen GA, Lawrence RL, Engel R, Long DS (2002) Wheat yield estimates using multi-temporal NDVI satellite imagery. International Journal of Remote Sensing 23: 4169–4180.

[24] Kastens JH, Kastens TL, Kastens DLA, Price KP, Martinko EA, et al (2005) Image masking for crop yield forecasting using AVHRR NDVI time series imagery. Remote Sensing of Environment 99: 341–356.

[25] Kalubarme AH, Potdar MB, Manjunath KR, Mahey RK, Siddhu SS (2003) Growth profile based crop yield models: a case study of large area wheat yield modelling and its extendibility using atmospheric corrected NOAA AVHRR data. International Journal of Remote Sensing 24: 2037–2054.

[26] Huang JF, Wang RC, Wang XZ, Liu SM, Zhang JH (1999) Study on multiple yield estimation models of winter wheat using remote sensing data. Journal of Zhejiang University (Agric & Life Sci) 25: 519–523 (in Chinese with English abstract)..

[27] Hochheim KP, Barber DG (1998) Spring wheat yield estimation for Western Canada using NOAA NDVI data. Canadian Journal of Remote Sensing 24: 17–27.

[28] Gupta R, Prasad S, Rao G, Nadham T (1993) District level wheat yield estimation using NOAA/AVHRR NDVI temporal profile. Advances in Space Research 13: 253–256.

[29] Ferencz C, Bognar P, Lichtenberger J, Hamar D, Tarscai G, et al (2004) Crop yield estimation by satellite remote sensing. International Journal of Remote Sensing 25: 4113–4149.

[30] Dubey RP, Ajwani N, Kalubarme MH, Sridhar VN, Navalgund RR, et al (1994) Preharvest wheat yield and production estimation for the Punjab, India. International Journal of Remote Sensing 15: 2137–2144.

[31] Doraiswamy PC, Cook PW (1995) Spring wheat yield assessment using NOAA AVHRR data. Canadian Journal of Remote Sensing 21: 43–51.

[32] Cheng Q (1994) The use of vegetation index for monitoring drought and winter wheat yield estimation. Remote sensing technology and application 9: 12–18 (in Chinese with English abstract)..

[33] Bullock PR (1992) Operational estimates of western Canadian grain production using NOAA AVHRR LAC data. Canadian Journal of Remote Sensing 18: 23–29.

[34] Boken VK, Shaykewich CF (2002) Improving an operational wheat yield model using phenological phase-based Normalized Difference Vegetation Index. International Journal of Remote Sensing 23: 4155–4168.

[35] Benedetti R, Rossini P (1993) On the use of NDVI profiles as a tool for agricultural statistics: the case study of wheat yield estimate and forecast in Emilia Romagna. Remote Sensing of Environment 45: 311–326.

[36] Becker-Reshef I, Vermote E, Lindeman M, Justice C (2010) A generalized regression-based model for forecasting winter wheat yields in Kansas and Ukraine using MODIS data. Remote Sensing of Environment 114: 1312–1323.

[37] Bastiaanssen WGM, Ali S (2003) A new crop yield forecasting model based on satellite measurements applied across the Indus Basin, Pakistan. Agriculture Ecosystems & Environment 94: 321–340.

[38] Balaghi R, Tychon B, Eerens H, Jlibene M (2008) Empirical regression models using NDVI, rainfall and temperature data for the early prediction of wheat grain yields in Morocco. International Journal of Applied Earth Observation and Geoinformation 10: 438–452.

[39] Wannebo A, Rosenzweig C (2003) Remote sensing of US cornbelt areas sensitive to the El Nino-Southern Oscillation. International Journal of Remote Sensing 24: 2055–2067.

[40] Unganai LS, Kogan FN (1998) Drought monitoring and corn yield estimation in Southern Africa from AVHRR data. Remote Sensing of Environment 63: 219–232.

[41] Seiler RA, Kogan F, Wei G (2000) Monitoring weather impact and crop yield from NOAA AVHRR data in Argentina. In: Gupta RK, editor. Remote Sensing for Land Surface Characterisation. 1177–1185.

[42] Rojas O (2007) Operational maize yield model development and validation based on remote sensing and agro-meteorological data in Kenya. International Journal of Remote Sensing 28: 3775–3793.

[43] Reynolds CA, Yitayew M, Slack DC, Hutchinson CF, Huete A, et al (2000) Estimating crop yields and production by integrating the FAO Crop specific Water Balance model with real-time satellite data and ground-based ancillary data. International Journal of Remote Sensing 21: 3487–3508.

[44] Quarmby N, Milnes M, Hindle T, Silleos N (1993) The use of multi-temporal NDVI measurements from AVHRR data for crop yield estimation and prediction. International Journal of Remote Sensing 14: 199–210.

[45] Mkhabela MS, Mashinini NN (2005) Early maize yield forecasting in the four agro-ecological regions of Swaziland using NDVI data derived from NOAAs-AVHRR. Agricultural and Forest Meteorology 129: 1–9.

[46] Lewis JE, Rowland J, Nadeau A (1998) Estimating maize production in Kenya using NDVI: some statistical considerations. International Journal of Remote Sensing 19: 2609–2617.

[47] Lee R, Kastens D, Price K, Martinko E. Forecasting corn yield in Iowa using remotely sensed data and vegetation phenology information; 2000 January 10–12; Lake Buena Vista, Florida. 460–467.

[48] Hayes M, Decker W (1996) Using NOAA AVHRR data to estimate maize production in the United States Corn Belt. International Journal of Remote Sensing 17: 3189–3200.

[49] Funk C, Budde ME (2009) Phenologically-tuned MODIS NDVI-based production anomaly estimates for Zimbabwe. Remote Sensing of Environment 113: 115–125.

[50] Tennakoon SB, Murty VVN, Eiumnoh A (1992) Estimation of cropped area and grain-yield of rice using remote-sensing data. International Journal of Remote Sensing 13: 427–439.

[51] Huang JF, Yang ZE, Wang RC, Xu HW, Jiang HX (2002) The rice production forecasting models using NOAA/AVHRR data based on GIS. Remote sensing technology and application 17: 125–128 (in Chinese with English abstract)..

[52] Huang JF, Wang FM, Wang XZ (2010) Hyperspectral experiment for paddy rice remote sensing; Huang JQ, Chen JY, editors. Hangzhou: Zhejiang University Press. 315 p. (in Chinese with English abstract).

[53] Rasmussen MS (1998) Developing simple, operational, consistent NDVI-vegetation models by applying environmental and climatic information: Part I. Assessment of net primary production. International Journal of Remote Sensing 19: 97–117.

[54] Rasmussen MS (1997) Operational yield forecast using AVHRR NDVI data: Reduction of environmental and inter-annual variability. International Journal of Remote Sensing 18: 1059–1077.

[55] Rasmussen MS (1992) Assessment of millet yields and production in northern Burkina Faso using integrated NDVI from the AVHRR. International Journal of Remote Sensing 13: 3431–3442.

[56] Maselli F, Romanelli S, Bottai L, Maracchi G (2000) Processing of GAC NDVI data for yield forecasting in the Sahelian region. International Journal of Remote Sensing 21: 3509–3523.

[57] Groten SME (1993) NDVI – Crop Monitoring and Early Yield Assessment of Burkina-Faso. International Journal of Remote Sensing 14: 1495–1515.

[58] Potdar MB (1993) Sorghum yield modelling based on crop growth parameters determined from visible and near-IR channel NOAA AVHRR data. International Journal of Remote Sensing 14: 895–905.

[59] Fuller DO (1998) Trends in NDVI time series and their relation to rangeland and crop production in Senegal, 1987–1993. International Journal of Remote Sensing 19: 2013–2018.

[60] Wendroth O, Reuter HI, Kersebaum KC (2003) Predicting yield of barley across a landscape: a state-space modeling approach. Journal of Hydrology 272: 250–263.

[61] Weissteiner C, Kühbauch W (2005) Regional Yield Forecasts of Malting Barley (Hordeum vulgare L.) by NOAA-AVHRR Remote Sensing Data and Ancillary Data. Journal of agronomy and crop science 191: 308–320.

[62] Liu WT, Kogan F (2002) Monitoring Brazilian soybean production using NOAA/AVHRR based vegetation condition indices. International Journal of Remote Sensing 23: 1161–1179.

[63] Esquerdo J, Zullo J, Antunes JFG (2011) Use of NDVI/AVHRR time-series profiles for soybean crop monitoring in Brazil. International Journal of Remote Sensing 32: 3711–3727.

[64] Tucker CJ (1979) Red and photographic infrared linear combinations for monitoring vegetation. Remote Sensing of Environment 8: 127–150.

[65] Tilman D, Cassman KG, Matson PA, Naylor R, Polasky S (2002) Agricultural sustainability and intensive production practices. Nature 418: 671–677. 12167873 10.1038/nature01014

[66] Evenson RE, Gollin D (2003) Assessing the impact of the Green Revolution, 1960 to 2000. Science 300: 758. 12730592 10.1126/science.1078710

[67] Peng S, Laza R, Visperas R, Sanico A, Cassman KG, et al (2000) Grain yield of rice cultivars and lines developed in the Philippines since 1966. Crop Science 40: 307–314.

[68] Hafner S (2003) Trends in maize, rice, and wheat yields for 188 nations over the past 40 years: a prevalence of linear growth. Agriculture, ecosystems & environment 97: 275–283.

[69] Pinzon J, Brown ME, Tucker CJ (2004) Satellite time series correction of orbital drift artifacts using empirical mode decomposition. Hilbert-Huang Transform: Introduction and Applications 10: 285–295.

[70] Tucker CJ, Pinzon JE, Brown ME, Slayback DA, Pak EW, et al (2005) An extended AVHRR 8-km NDVI dataset compatible with MODIS and SPOT vegetation NDVI data. International Journal of Remote Sensing 26: 4485–4498.

[71] Fensholt R, Nielsen TT, Stisen S (2006) Evaluation of AVHRR PAL and GIMMS 10-day composite NDVI time series products using SPOT-4 vegetation data for the African continent. International Journal of Remote Sensing 27: 2719–2733.

[72] Fensholt R, Rasmussen K, Nielsen TT, Mbow C (2009) Evaluation of earth observation based long term vegetation trends – Intercomparing NDVI time series trend analysis consistency of Sahel from AVHRR GIMMS, Terra MODIS and SPOT VGT data. Remote Sensing of Environment 113: 1886–1898.

[73] Genovese G, Vignolles C, Negre T, Passera G (2001) A methodology for a combined use of normalised difference vegetation index and CORINE land cover data for crop yield monitoring and forecasting. A case study on Spain. Agronomie 21: 91–111.

[74] Freund JT (2005) Estimating Crop Production in Kenya: A Multi-Temporal Remote Sensing Approach [Masters]. Santa Barbara: University of California. 59 p.

[75] Tucker CJ, Holben BN, Elgin JH, McMurtrey JE (1980) Relationship of spectral data to grain yield variation. Photogrammetric Engineering and Remote Sensing 46: 657–666.

[76] Dyson T (1999) World food trends and prospects to 2025. Proceedings of the National Academy of Sciences 96: 5929. 10.1073/pnas.96.11.5929 PMC34208 10339520

[77] Clarke FR, Baker RJ, Depauw RM (1994) Moving mean and least-squares smoothing for analysis of grain-yield data. Crop Science 34: 1479–1483.

[78] Peltonen-Sainio P, Jauhiainen L, Laurila IP (2009) Cereal yield trends in northern European conditions: Changes in yield potential and its realisation. Field Crops Research 110: 85–90.

[79] Epplin FM, Peeper TF (1998) Influence of planting date and environment on Oklahoma wheat grain yield trend from 1963 to 1995. Canadian Journal of Plant Science 78: 71–77.

[80] Stergiou KI, Christou ED (1996) Modelling and forecasting annual fisheries catches: Comparison of regression, univariate and multivariate time series methods. Fisheries Research 25: 105–138.

[81] Foody GM, Boyd DS, Cutler MEJ (2003) Predictive relations of tropical forest biomass from Landsat TM data and their transferability between regions. Remote Sensing of Environment 85: 463–474.

[82] Sirkin RM (2006) Statistics for the social sciences. Thousand OaksCalifornia: Sage Publications, Inc. 244 p.

